# Generating patient-specific computational models with point cloud data from human atrial electrophysiology studies

**DOI:** 10.1371/journal.pone.0344274

**Published:** 2026-03-26

**Authors:** Josue Nataren Moran, Laryssa Abdala, Boyce E. Griffith

**Affiliations:** 1 Department of Biomedical Engineering, Duke University, Durham, North Carolina, United States of America; 2 Department of Mathematics, University of North Carolina, Chapel Hill, North Carolina, United States of America; 3 Department of Biomedical Engineering, University of North Carolina, Chapel Hill, North Carolina, United States of America; 4 Carolina Center for Interdisciplinary Applied Mathematics, University of North Carolina, Chapel Hill, North Carolina, United States of America; 5 Computational Medicine Program, University of North Carolina School of Medicine, Chapel Hill, North Carolina, United States of America; 6 McAllister Heart Institute, University of North Carolina School of Medicine, Chapel Hill, North Carolina, United States of America; University of Southern California, UNITED STATES OF AMERICA

## Abstract

One in five patients diagnosed with atrial fibrillation die within a year after being diagnosed according to a prospective cohort study done in the United Kingdom, making this disease the focus of many scientific studies. One approach to studying this disease has been computational models since they have demonstrated powerful capabilities in understanding and analyzing the biochemical processes underlying atrial fibrillation. To create accurate patient models for studying cardiovascular diseases, we developed a pipeline for generating patient-specific models of the left atrial posterior wall using point cloud data without image segmentation. Our goal was to evaluate the performance of these models by comparing simulated electrograms to those obtained from atrial fibrillation patients. We created models for two different paroxysmal atrial fibrillation patients under healthy tissue conditions. To validate our model, we compared simulated and measured electrograms using various metrics. Some electrograms matched well in terms of local activation time and cross-correlation peak, whereas others showed significant differences in amplitude and duration. Additionally, we explored the impact of modeling fibrotic tissue on electrogram morphology by creating four persistent atrial fibrillation patient models with varying fibrosis densities and types. Simulations indicated that increased modeled fibrosis density led to more multiphasic electrogram morphologies, with little impact from fibrosis type. The fibrosis simulations also had morphological characteristics seen in other fibrosis electrophysiology modeling studies like deflections patterns and amplitudes, strengthening the reasoning behind using this type of model generation methodology. Our findings suggest that point cloud data is sufficient for creating accurate left atrial posterior wall models, which can simulate electrograms comparable to measured waveforms. This method could be useful for patient-specific studies, potential specialized ablation procedures, and arrhythmia research.

## 1 Introduction

Cardiovascular diseases cause the majority of deaths each year worldwide [1]. Computational models have proved to be useful tools in understanding and developing diagnostic and therapeutic platforms that allow for repeatability, variability, and a safe-for-patients methodology [2]. From lumped parameter (zero-dimensional) models used to lump whole vessels and heart chambers into single components with flow rates and pressures [3,4] to one- and two-dimensional computational models used to describe tissue dynamics in space and time [5,6], computational models have been widely used in cardiac studies to advance our physiology knowledge and clinical applications. Usually, for more detailed patient-specific models, three-dimensional modeling is preferred since most physiological behaviors have three-dimensional spatial influence. This type of modeling is also preferred due to the obtention of medical images and signals that can be acquired for patients in the clinic [7,8]. Typically, medical images are the starting point to creating patient-specific models, since these images are segmented and then models can be created [9,10].

Electroanatomical mapping systems (EAMS) like CARTO 3 and EnSite X are used in the clinic to guide ablations and obtain important patient biological data by recording electrograms (EGMs) and point cloud data (PCD) [11,12]. Typically, computed tomography (CT) and magnetic resonance (MR) imaging are used along with electroanatomical recordings for patient-specific modeling outside of these EAMS [13], although the access of such data is not a straightforward task due to the nature of the proprietary data format the data is exported in for which efforts have been made to create tools for accessing EAMS data [14]. The data in the EAMS integrates well with the proprietary software that created the source patient data, and it provides tools ready to be used like mathematical meshes and geometric visualization; but the version of this data being exported from these systems cannot be readily used in simulation studies unless using closed-source software. Since the exported data from the EAMS is not in a ready-to-be-use format suitable for *in silico* studies, additional model generation methods are always needed in order to create the input data for modeling studies. In addition, additional steps are required to create volumetric computational meshes from surface meshes coming from EAMS; having three-dimensional models is highly relevant when studies focus on the effect of factors that can only be assessed with three-dimensional geometries like when studying muscle thickness and curvature effect in cardiac wave propagation [15]. Attempts have also been made to use PCD without medical images combined with machine learning approaches to generate physiologically accurate meshes [16,17]. However, many of these studies have focused primarily on geometric validation using imaging data or previously segmented meshes, and have not thoroughly assessed the electrophysiological functionality of the resulting geometries through recorded electrograms. Validated models are particularly relevant in persistent atrial fibrillation (AF) cases, where one of the main components is the electrophysiological behavior of the left atrial posterior wall (LAPW) [18]. Electrograms recorded from the LAPW are important since their morphologies and characteristics typically guide therapies, as it was done in the work by Xu et al. [19], in which they applied local pacing to evaluate pathological complex EGMs. To the best of our knowledge, there have not been any studies in which PCD with EGM waveforms validation is used to generate models without using medical images before the present work. This lack of studies might be attributed to the complex and diverse geometries of the LA.

PCD models could help in developing powerful, versatile, and compact tools to study complex tissue-reentry relationships. For instance, atrial fibrosis has been conventionally believed to be one of the most important factors to give rise and sustain AF [20]. On the other hand, recent clinical work suggests that atrial fibrosis may not be a hallmark of AF [21]. Specifically, Ramos and colleagues found that there was no strong correlation between fibrosis markers and electrophysiological abnormalities in data. In contrast, computational studies have shown the relevance of both fibrosis type and degree in eliciting and sustaining AF [22,23]. Consequently, at present, interactions between fibrosis type and degree and AF are poorly understood.

In this work, we created patient-specific LAPW PCD models of AF patients and evaluated their performance. The generated models were created directly from electrical anatomical mapping system data without any additional image segmentation. Patient-specific models of the LAPW were created for a total of six patients: two patient models had healthy tissue configuration while the other four had fibrotic tissue configurations. The scope of this study was limited to the LAPW surface due to its relatively simpler geometry as well as its high relevance in AF dynamics, particularly in persistent AF [18,24]. Model validation was done by comparing recorded and simulated EGMs and their characteristics using the healthy tissue models. Fibrosis patterns were generated into the other four PCD patient-specific models as a test-case of the use of PCD models to study atrial fibrosis degree and type effect on waveform characteristics.

## 2 Materials and methods

### 2.1 Patient data acquisition

Anonymized patient data coming from studies focusing on determinants of bipolar EGMs amplitude and conduction velocity maps using EGMs were used [25,26]. The data were obtained, identified, and accessed under the approval of the Duke Health Institutional Review Board (IRB protocol number 00032732). These data were accessed and utilized from March 1^st^ 2023 until December 31^st^ 2024, and no information that could potentially link the data to patients was accessed at any point during the study. In this dataset, EGMs and LAPW surface coordinate PCD were available for five paroxysmal AF and five persistent AF patients. These groups will be referred to as H1 and H2 groups, respectively, henceforth. These patient EGMs were recorded after pulmonary vein isolation procedures with no other complex ablation in the LAPW. High-density maps of the wall surface were obtained via CARTO 3 along with unipolar electrograms at a 1 kHz rate and a bandpass filter of 2–240 Hz. Patients were paced near the coronary sinus creating plane wave propagation patterns along the LAPW, so the EGMs did not reflect fibrillation. The first 140 msec of the waveforms were used to obtain only the propagation of the depolarization wave. Each patient is referred here as a combination of the group they belonged to (H1 or H2) and a letter representing the patient (e.g., the third patient of the persistent AF group was H2C). Data for patients H1A and H1B were used for modeling pipeline evaluation, whereas data for patients H2B, H2C, H2D, and H2E were used to explore the effect of fibrosis on waveform characteristics. The remaining four patients were excluded because their PCDs did not yield a robust surface reconstruction. Specifically, these PCDs were sparse and noisy, which prevented the generation of a continuous and anatomically plausible LAPW surface. Because we did not have access to the original CARTO data, we could not attempt to reprocess or denoise these datasets to improve the geometry representation. The electrograms and geometries, along with models and useful MATLAB scripts utilized for this work, can be found on a public repository [27].

### 2.2 Model construction

The general approach on the development of patient-specific models was taken from previous work similar to Rossi et al. [28]. The main difference between that prior work and the present study is our use of point cloud data points to create the initial wall surfaces without any image segmentation. This difference in methodology is significant because image segmentation has typically been considered the standard for patient-specific modeling [29]. For paroxysmal AF patients, isolating the reentry-inducing electrical activity from the pulmonary veins can be enough for treating AF [30]; in contrast, these procedures might not be enough for persistent AF patients because the LAPW becomes an important region of the atrium where reentry occurs and sustaining of AF happens [24]. Because of its importance and relatively simpler geometry compared to the bulk of the chamber, our objective was to generate models of the isolated LAPW without any image segmentation.

To create each model, we first obtained surface meshes for each patient’s LAPW using custom-made software in MATLAB. These surfaces were generated from the extracted LAPW geometry points in the PCD obtained from each patient. The initial approach was to turn the PCD points directly to a surface using the function pc2surfacemesh from the Lidar toolbox in MATLAB that uses a *ball-pivot* method to generate surfaces from PCD [31], but they were discarded since the resulting meshes had overlapping surfaces. Because the PCD was dense, the function generated surfaces that had very irregular element surface normal vectors, complicating the extrusion process. In classic image segmentation for mesh generation, extruding a surface is the typical approach to generate volumetric meshes. Because of the extrusion process, the surface normal vectors of neighboring elements’ surfaces need not to cause overlapping surfaces and problematic regions after extrusion, which was happening in these models when using the original PCD’s. To solve the issue with overlapping surfaces, each patient’s PCD was downsampled. This process creates a sparser representation of the geometries by selectively removing points, ensuring that the remaining points maintain a minimum distance from their nearest neighbors. The result is a more efficient representation of the original data while preserving essential geometric features. This process was done using the function pcdownsample from the Computer Vision toolbox of MATLAB, which resulted in an average distance of 3.5 mm between points instead of the original 1 mm average distance. After downsampling, the less dense cloud was turned into smooth surfaces using the ball-pivot method, which avoids creating overlapping geometries because of the reduction in the variability in local surface normals. This downsampled spacing provided stable meshes while preserving anatomical fidelity and is consistent with the resolution of late gadolinium enhancement MRI voxels (≈1–3  mm) and typical electrode spacing in electroanatomic mapping systems (≈2–6 mm) [32–36]. Downsampling had a smoothing effect that reduced redundancy without altering key atrial dimensions or wall curvature while preserving geometric features.

Once the surface mesh was smooth, the surface was exported in STL format using the function writeSurfaceMesh of the Lidar toolbox in MATLAB. From there, the exported surface was manipulated and cleaned in Blender [37] by eliminating problematic points that caused overlapping surfaces when extruding. The points leading to overlapping surfaces can be thought of as noise in the PCD for which the cleaning was necessary only to ensure smoothness of the surface. These remaining problematic points were very minimal (less than five points per case that needed manual removal), and some geometries did not have any problematic points. After cleaning the surfaces, they were extruded to a thickness of 2.3 mm—the thickness that LAPWs typically have [38]—in Blender in their element normal directions.

After the volume was exported from Blender in STL format, each volume was then meshed in Coreform Cubit using tetrahedral elements and exported in ExodusII format [39]. The resulting meshes were not fine enough for electrophysiology simulations convergence, therefore they were uniformly refined twice using custom-made software based on the C++ finite element library libMesh [40]. The resulting element sizes, between 200–300 microns, as it has been used in other studies [41]. Using these different steps, the meshes needed for computational modeling for each patient were created. A function found in the data repository already mentioned can be used to extract points of interest of new PCD for the interested user (Extraction_LAPW.m).

### 2.3 Muscle fibers and electrical anisotropy

To simulate anisotropy using the monodomain equations, we employ an anisotropic conductivity tensor. This tensor is expressed in a local coordinate system representing the muscle fiber, sheet, and cross-fiber directions.

Muscle fibers in the LAPW vary slightly in the transmural direction, following the septopulmonary and septo-atrial bundles [42]. This variation in muscle fibers makes the electrical propagation of action potentials nontrivial, emphasizing the need for correctly generated muscle fiber models to more accurately describe a patient’s electrophysiology. In our study, we associate a fiber coordinate system with each patient-specific mesh by mapping fields from a template mesh. The template anatomy incorporates fiber fields created using a rule-based method [28]. We map fiber orientations from the template to each patient-specific model using as translation and scaling, aligning the template mesh with the general geometry and node locations of the new LAPW mesh. The alignment was done for accurate fiber mapping from the template mesh to the patient-specific model; this type of mapping is consistent with multiple studies that suggest that LAPW muscle fibers exhibit consistent patterns across samples, justifying our atlas-based mapping approach [42–45], making this independent of how the surfaces were smoothed and how models were created in the first place.

The mapping of the fiber orientations from the template mesh to each patient-specific model was done by transformations like translation and scaling done to the template mesh to match the general geometry and locations of the nodes of the new LAPW mesh. This mapping included orthotropic conductivity values (three different directions). These mapped vectors were then used to define the conductivity tensors in the monodomain equations. The original conductivity values used were taken from Henriquez [46] (fiber direction: intracellular = 1.74 mS/cm and extracellular = 6.25 mS/cm; sheet and sheet-normal directions: intracellular = 0.193 mS/cm and extracellular = 2.36 mS/cm). As these are bidomain model conductivity values, the base monodomain conductivity in each direction was obtained using $\begin{array}{c} \frac{\sigma_{i}\sigma_{e}}{\sigma_{i}+\sigma_{e}} \end{array}$, which is half of the harmonic mean of $\begin{array}{c} \sigma_{i} \end{array}$ and $\begin{array}{c} \sigma_{e} \end{array}$ [47]. These conductivities were then scaled uniformly to match the patient-specific conduction velocity (CV) calculated from the measured electrograms, so each patient had different conductivity values. Conduction velocity was calculated by dividing the total distance travelled by the propagation wave by activation times difference from points towards the beginning and end of propagation. For the scaling of the intracellular and extracellular conductivities to calculate the monodomain conductivities, the initial conductivities from Henriquez [46] were scaled iteratively based on comparison of the macro CV measurements (in cm/s) of the simulation results to the patient electrode paced CV. This was done via [48]:


$$\begin{array}{c} CV\propto \sqrt{\frac{\sigma_{i}\sigma_{e}}{\sigma_{i}+\sigma_{e}}}\approx K\sqrt{\frac{\sigma_{i}\sigma_{e}}{\sigma_{i}+\sigma_{e}}} \end{array}$$ (1)

The factor $\begin{array}{c} \text{K} \end{array}$ was calculated based on the longitudinal (fiber) electrical propagation, and the same factor was used to scale the conductivities in the transversal direction. An anisotropic conductivity tensor was used, but all components were uniformly scaled using a single measured conduction velocity, as propagation occurred mainly along one direction.

### 2.4 Stimulus sites

**Fig 1:**
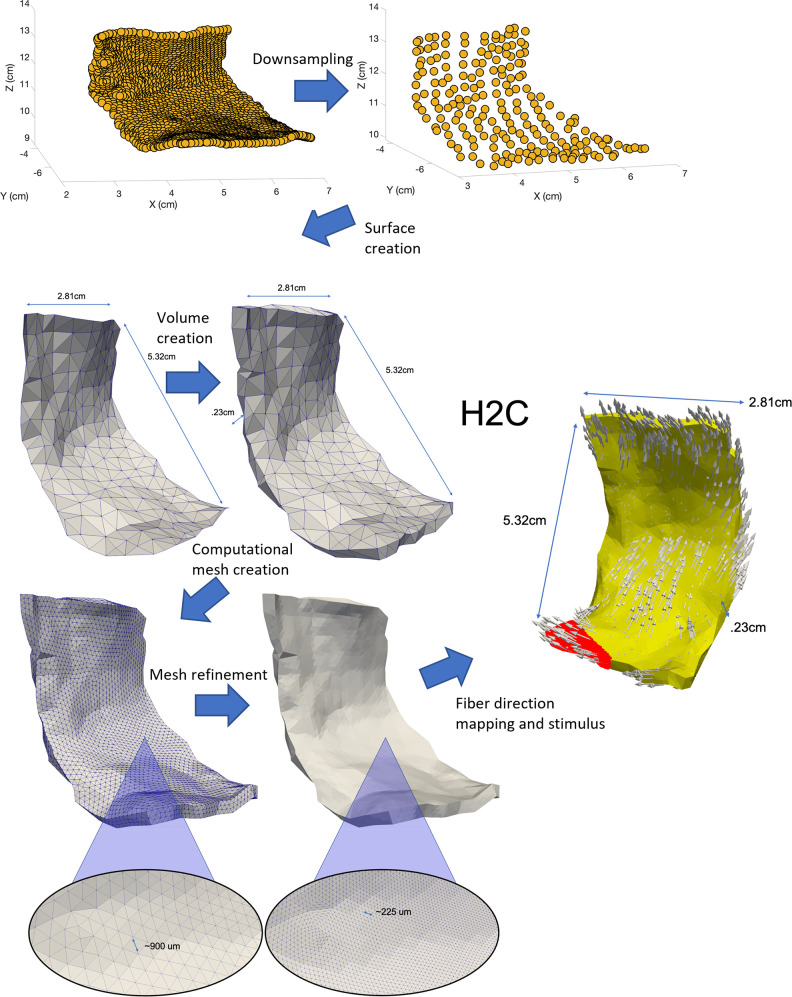
Point Cloud Data (PCD) Model Generation. The several steps to generate left atrial posterior wall (LAPW) PCD models are shown. First, the extracted LAPW points are downsampled. After downsampling, a surface mesh is created. This surface mesh is then extruded a specific thickness (2.3 mm in this work) to create a volume representing the LAPW. This volume is then meshed and refined to solve electrophysiology models in it. After mapping fiber orientations and determining stimulus sites, the model can be used along with other mathematical models such as the monodomain and Courtemanche models to simulate cardiac electrical propagation.

**Fig 2:**
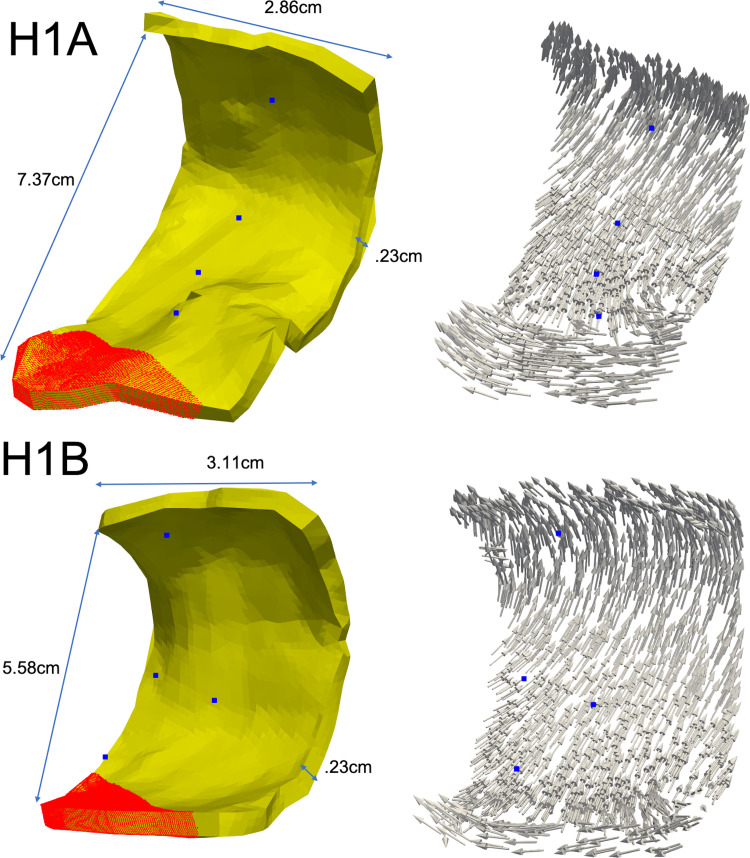
Patients H1A and H1B Models. The 3D geometry shows dimensions with fiber orientations for both patient H1A (left) and H1B (right). Blue points are electrode locations in space and red points in the geometry are points that were stimulated. These patient models did not have any atrial fibrosis added to their tissue substrates.

After determining fiber orientations for each model, stimulus sites to start a plane wave propagation pattern needed to be determined. Since in the original clinical procedures the atria were stimulated close to the coronary sinus, tissue points that were stimulated first in the patients’ procedures were outside the scope of the LAPW. To determine stimulus points that could generate a similar pattern to the original propagation sequence, local activation times (LATs) obtained from the recorded EGMs were evaluated for each patient to determine regions with the earliest activation times [49]. For both the simulated and measured waveforms, LATs were defined as the time point when $\begin{array}{c} \text{d}V_{\text{m}}/\text{d}t \end{array}$ was at its minimum value [25]. Tissue points closest to the electrodes that had EGMs LATs with 2 msec of earliest activation were selected as stimulus nodes in the geometry. A cube containing the points to be stimulated (i.e., all the points contained within the cube were selected as stimulus locations) was determined by selecting minimum and maximum coordinate points to enclose the region of the LAPW. Stimulation was done by producing an artificial current in the governing equations that elicited the start of the propagation. At these chosen nodes, a stimulus strength of 25 pA/pF for 2.5 msec was applied, and not as volumetric currents since when using monodomain models this is a more challenging approach. Fig 1 shows the model construction steps from geometry construction to stimulus site determination, and Fig 2 shows two final sample models for patients H1A and H1B used in the validation of the model-generation pipeline.

### 2.5 Electrophysiological model

This study used the monodomain model of electrical propagation with transmembrane ion dynamics determined by the Courtemanche model to simulate cardiac electrical activation and propagation [50]. The following are the relevant equations:


$$\begin{array}{c} \chi \left(C_{\text{m}}\frac{\partial V_{\text{m}}}{\partial t}(\underset{―}{x},t)+I_{\text{ion}}(\underset{―}{x},t)+I_{\text{stim}}(\underset{―}{x},t)\right)=\nabla \cdot {\underset{―}{\underset{―}{\sigma }}}_{\text{m}}\nabla V_{\text{m}}\left(\underset{―}{x},t\right),\;\underset{―}{x}\in \Omega_{\text{tissue}}, \end{array}$$ (2)

$$\begin{array}{c} {\underset{―}{\underset{―}{\sigma }}}_{\text{m}}=\sigma_{l}\underset{―}{f}⊗\underset{―}{f}+\sigma_{t}\left(\underset{―}{\underset{―}{I}}-\underset{―}{f}⊗\underset{―}{f}\right), \end{array}$$ (3)

$$\begin{array}{c} \sigma_{\text{l}}=\frac{\sigma_{\text{l},\text{i}}\sigma_{\text{l},\text{e}}}{\sigma_{\text{l},\text{i}}+\sigma_{\text{l},\text{e}}},\;\sigma_{\text{t}}=\frac{\sigma_{\text{t},\text{i}}\sigma_{\text{t},\text{e}}}{\sigma_{\text{t},\text{i}}+\sigma_{\text{t},\text{e}}}, \end{array}$$ (4)

$$\begin{array}{c} \phi_{\text{e}}(\underset{―}{x},t)=\frac{\text{1}}{\text{4}\pi \sigma_{\text{b}}}\int \Omega_{\text{tissue}}\frac{\chi I_{\text{m}}(\underset{―}{x},t)}{\left\|\underset{―}{x}-{\underset{―}{x}}^{\prime }\right\|}\text{d}{\underset{―}{x}}^{\prime }, \end{array}$$ (5)

in which $\begin{array}{c} \sigma_{\text{l},\text{e}},\sigma_{\text{t},\text{e}},\sigma_{\text{l},\text{i}},\text{ and }\sigma_{\text{t},\text{i}} \end{array}$ corresponds to the longitudinal and transversal conductivities of the intracellular and extracellular compartments, ${\underset{―}{\underset{―}{\sigma }}}_{\text{m}}$ is the functional monodomain conductivity tensor, and $\begin{array}{c} \sigma_{\text{b}} \end{array}$ is the blood bath conductivity, $C_{\text{m}}$ is the membrane capacitance (here 1 $\begin{array}{c} \mu \text{F}/\text{c}{\text{m}}^{\text{2}} \end{array}$), χ is the cell membrane area per unit volume of tissue $\begin{array}{c} (\text{here as }\text{1},\text{500 }{\text{cm}}^{-\text{1}}),\left\|\underset{―}{x}-{\underset{―}{x}}^{\prime }\right\| \end{array}$ is the distance from the electrode point to each source, and $V_{\text{m}}$ is the transmembrane potential difference (in mV). Equations 2 and 3 are the monodomain model. Equation 4 shows how each conductivity tensor was formulated as a summation of the individual contributions of the fiber, sheet, and sheet-normal direction conductivities. For each direction, the vector tensor product for each individual direction was scaled by the conductivity value in that direction. Equation 5 is used to determine the extracellular signals or EGMs from the transmembrane current per unit membrane area, $I_{\text{m}}=C_{\text{m}}\frac{\partial V_{\text{m}}}{\partial t}+I_{\text{ion}}+I_{\text{stim}}$. With this approach, the extracellular potential at arbitrary virtual electrodes depends on the superposition of many different sources from the tissue nodes, the distance between the sources and the virtual electrode recording waveforms, and the blood bath properties representing the medium conductivity [47]. The total ionic current in these equations is broken down into a series of individual currents based on the different ions participating in the action potential generation (i.e., potassium, sodium, calcium) as well as pump currents and calcium-induced calcium-release currents. For the detailed description of these equations, refer to Courtemanche et al. [50]. No-flux boundary conditions were applied for the simulations.

The finite element method was used to discretize the equations in space using linear lagrangian polynomial interpolation with first order elements [2], and an explicit time integrator was used [51] with a 0.001 msec time discretization for the monodomain simulations. Custom scripts were implemented leveraging libMesh’s powerful functions and tools to solve for these partial differential equations. The scripts had the capability to load the mesh, perform mesh refinement, set the system of equations, create the forcing vectors and relevant stiffness and mass matrices, and solve the system of equations. The libMesh library was used as engine to perform all of these steps, as well as for parallelizing the simulation run using libMesh’s support for MPI communication. Simulations were run on the Dogwood high-performance computer cluster at the University of North Carolina at Chapel Hill, which provides a computing cores connected via a low-latency, high-bandwidth network. The simulated EGMs obtained from the simulations were downsampled to 1 kHz and filtered between 2 and 220 Hz, which were the settings that the original waveforms reflected. The time downsampling was done because we first compute high-resolution EGMs in parallel with the capturing of membrane current dynamics using Courtemanche model, which requires high time resolution, but then downsample them to match the lower temporal resolution of clinical recordings.

### 2.6 Comparison metrics

To evaluate the performance of the models, the recorded waveforms were compared to the waveforms coming from the simulations. This comparison was done by waveform characterization and by comparing the characteristic parameters of the waveforms of each group using different comparison approaches. The methods used to compare such parameters were calculating Cohen’s d values for the characteristic parameters, evaluating the spatial difference of the waveforms in the principal component space, as well as graphical methods using isosurfaces that are explained later.

Waveform characterization was done for each EGM as a first step for most of the approaches used. The following characteristic parameters were obtained for each EGM: the downstroke (maximum negative $\begin{array}{c} \text{d}V_{\text{m}}/\text{d}t \end{array}$), rising upstroke (maximum $\begin{array}{c} \text{d}V_{\text{m}}/\text{d}t \end{array}$ of the positive deflection), recovery upstroke (maximum $\begin{array}{c} \text{d}V_{\text{m}}/\text{d}t \end{array}$ after the negative deflection), signal width (time length between absolute maximum and minimum peaks), EGM duration (duration of the whole signal including the whole of the positive and negative deflections), baseline (average baseline of the waveform), peak-to-peak amplitude, and deflection count (number of deflections in the waveform) [52–56].

Using the characteristic parameters mentioned above, Cohen’s d values for each recorded-simulated EGM pair were obtained for each parameter [57]. This metric was evaluated to quantify the effect size of the difference between what was observed in the recorded and simulated EGM pairs [57]. This method uses the sizes of the populations being evaluated (recorded and simulated electrograms) and the mean and standard deviation of the parameters of both groups. The mean difference of the two populations is divided by a pooled standard deviation to obtain Cohen’s d values. If this d value is less than 0.2, it is typically taken as the effect size of the difference being very small or negligible. If the d value is between 0.2 and 0.5 the effect size is taken as small but noticeable, and if the d value is between 0.5 and 0.8 the effect size is taken as medium and relevant. If the d value is greater than 0.8 it is interpreted as the effect size being large implying that the difference between the means is very significant. The following equations detail the computation of the Cohen’s d value, with $SD_{\text{pooled}}$ being the pooled standard deviation, n being the number of EGMs for each group (which was always the same for both the recorded and simulated waveforms), SD being the parameter standard deviation of each group, and M being the parameter mean of each group:


$$SD_{\text{pooled}}=\sqrt{\frac{\left(n_{\text{recorded}}-\text{1}\right)S\overset{\text{2}}{\underset{\text{recorded}}{D}}+\left(n_{\text{simulated}}-\text{1}\right)S\overset{\text{2}}{\underset{\text{simulated}}{D}}}{n_{\text{recorded}}+n_{\text{simulated}}-\text{2}}}$$ (6)

$$d=\frac{M_{\text{recorded}}-M_{\text{simulated}}}{SD_{\text{pooled}}}$$ (7)

Average error values were also calculated for three of the characteristic parameters mentioned above that are typically more relevant in the clinical setting as well as error values for the LATs. The inclusion of LATs error was done to evaluate how well the models resembled the activation sequence seen from the recorded patient EGMs. Correlation coefficients and cross-correlation peaks averages were obtained as well using MATLAB built-in functions as a metric of how good the similarity between the traces were [55].

Principal component analysis (PCA) was used to reduce the variable space and to obtain a more graphical way of evaluating the models’ performance [58–60]. Before evaluating the PCs, wavelet decomposition of each normalized waveform was performed to obtain the detail coefficients describing each waveform. The normalized waveforms were used to obtain the detail coefficients since this comparison focused on signal morphology and not amplitude. Wavelet decomposition was used to minimize the number of relevant values describing the EGMs. Matrices containing the detail coefficients were obtained using a biorthogonal wavelet during the fourth-order wavelet decomposition for each recorded-simulated waveform pair of Patients H1A and H1B. This order of decomposition was used so that after applying PCA to the coefficient matrices the first five PCs were able to explain above 90% variability [61–63]. These PCs were then clustered using the k-means algorithm [64]. The principal components in the PCA space for each simulated waveform were also obtained using the eigenvectors to have simulated data presented in the same PC space along with recorded data. Seeing where signals lie in the PC space provides a qualitative description of how models perform since the closer the simulated data points are to the recorded data points helps visualizing how well a model describes physical phenomena. The distance from each waveform PCs in space to the cluster centroid was also evaluated to look at how different and spaced apart the simulated EGMs were in comparison to the recorded waveforms.

Isosurfaces were also created for the recorded and simulated data of patients H1A and H1B. Isosurfaces here refer to isochrone maps showing colored discrete ranges of EGMs LATs projected into the PCD surface LAPW points [65]. Electrode LATs from the EGMs were mapped onto the tissue surface points coming from the PCD based on proximity. The ranges of the colors were determined based on the recorded EGM’s activation times, to evaluate visually how the simulated data performed against the recorded waveforms. Because of this specific colormap ranges determination, this was specific for each patient based on the minimum and maximum LATs values.

### 2.7 Fibrosis

Atrial fibrosis was used to explore the usage of PCD models to gain insight into tissue properties and their relationship to EGM characteristics. In atrial fibrosis, fibrotic obstacles create non-conductive blockages that can cause abnormal electrical pathways that lead to arrhythmias [66]. There are four different fibrosis patterns observed commonly in the clinical setting: compact; diffuse; interstitial; and patchy [67]. Fibrogenesis is still a field of study as well as its effect in cardiac function, but it is believed that these different types of atrial fibrosis have different effects on cardiac propagation [68,69].

Researchers have modeled fibrosis using different approaches [22,54,70]. In this study, fibrosis was modeled using percolation methods that eliminate elements from the tissue mesh, creating holes and forcing propagation around these blockages since there have been studies that show this methodology yields clinically comparable data [70]. To create these holes when looping over all the mesh elements of each model and assigning the subdomains, a new subdomain tag was created for each node in which fibrosis was desired to be present. Transmembrane voltage and current were not computed in this subdomain [70].

A Perlin noise-based model designed by Lawson et al. [71] was used to generate the four different atrial fibrosis patterns. This method yields patterns comparable to what was seen in histological studies [67]. These fibrotic patterns built from discrete points in computational space can then be mapped onto a mesh to represent elements that belong to fibrotic tissue domains. We extended the 2D method by Lawson et al. into a 3D implementation. Consequently, the different harmonic fields used to create the fibrosis patterns were based on x, y and z coordinates. Additionally, instead of using randomly generated fiber fields, we use the fiber direction defined by the atlas-based method described above.

Using the Perlin noise methodology, the four different types of atrial fibrosis were simulated for four different patient geometries (H2B, H2C, H2D, and H2E). This modeling for the four different geometries was done at three different fibrosis densities of 10%, 35%, and 60%. These densities represented the proportion of points in the geometry that would be considered fibrotic elements meaning no action potential propagation would be elicited in them (implemented as holes in the geometry where the monodomain equations were not calculated). For each combination (atrial fibrosis type and atrial fibrosis degree) for each geometry, a total of ten different fibrosis patterns were generated. A total of 480 patterns were created to explore the characteristics and relevance of the different fibrotic tissue configurations or combinations representing different tissue substrates. Because simulations considering atrial fibrosis were expected to have more complex signals [72], not all characteristic parameters were used. We compared these waveforms coming from fibrotic substrates using the characteristics peak-to-peak amplitude, number of deflections, and EGM duration. Amplitude and EGM duration are important in the clinical setting because they can detect ablation targets by indicating where reentrant points might be occurring. Deflection count is also used to distinguish between complex and smooth signals. Average values and standard deviations for each parameter were obtained to describe the tissue substrates.

## 3 Results

### 3.1 Characteristic parameters and error averages

The characteristic parameters were used to compare the measured and simulated EGM signals. Table 1 shows the mean and standard deviation values for each of the characteristic parameters of the measured-simulated EGM pairs for both patients H1A and H1B. The EGM durations were higher in recorded waveforms compared to simulated waveforms. This could be a result of the patient tissue substrates having fibrosis, which is not included in these simulations. Baseline values (refering to the extracellular potential recorded in the absence of local action potential propagation) had near-zero mv values as expected. The Cohen’s d values are also reported in Table 1; cells in the table are shaded based on whether the effect size was very small, small, medium, or large. Only one characteristic parameter in patient H1B had a characteristic difference with very small effect size, with a few being small effect sizes. Based on the number of characteristic parameters that did not have large effect sizes, patient H1B model seems to have performed better compared to patient H1A model due to having a smaller average Cohen’s d value and having one less characteristic parameter with a d value above 0.8 than the other model.

**Table 1:** Waveform Characteristics Comparison. Summary of mean and standard deviation for all the characteristic parameters across all the signals is shown for both patient and simulated data for H1A and H1B. For both patients, the Cohen’s d value was calculated to measure the effect size of the recorded-simulated characteristics’ differences taking into account that there were 606 and 855 electrograms for patient H1A and H1B respectively. Typically, a Cohen’s d value smaller than 0.2 indicates an almost negligible effect size (unshaded in the table). Values between 0.2 and 0.5 indicate small but noticeable effect size (shaded light grey in the table), while values between 0.5 and 0.8 indicate moderate or medium effect size (shaded medium grey in the table). Cohen’s d values greater than 0.8 typically indicate very large effect size representing a significant disparity in this case between recorded and simulated electrogram characteristics (shaded dark grey in the table).

	H1A (patient)	H1A (simulation)	Cohen’s d (H1A)	H1B (patient)	H1B (simulation)	Cohen’s d (H1B)
Downstroke (mV/msec)	−0.37 ± 0.36	−0.3 ± 0.25	0.23	−0.82 ± 0.74	−1.52 ± 2.4	0.40
Rising Upstroke (mV/msec)	0.06 ± 0.13	0.13 ± 0.09	0.64	0.12 ± 0.16	0.33 ± 0.43	0.65
Recovery Upstroke (mV/msec)	0.14 ± 0.1	0.1 ± 0.09	0.38	0.33 ± 0.31	0.35 ± 0.55	0.04
Signal Width (msec)	63.46 ± 57.66	15.57 ± 6.33	1.17	30.51 ± 46.7	10.65 ± 6.3	0.60
Baseline (mV)	−0.07 ± 0.07	0.01 ± 0.02	1.55	−0.09 ± 0.09	0.01 ± 0.04	1.44
EGM Duration (msec)	110.23 ± 45.9	82.3 ± 10.08	0.84	107.39 ± 47.94	69.98 ± 10.5	1.08
Peak-Peak Amplitude (mV)	1.22 ± 0.85	1.9 ± 0.85	0.80	2.83 ± 1.77	4.4 ± 4.28	0.48
Deflection Count	5.86 ± 2.41	3.23 ± 0.81	1.46	4.47 ± 1.78	2.31 ± 0.57	1.64

An analysis of the lumped characteristic parameters based on error values was also performed and is reported in Table 2. Average error values for EGM duration, deflection count, peak-peak amplitude, and LATs were obtained for each recorded-simulated EGM pair, along with average correlation coefficient for the waveform traces and cross-correlation peaks for the normalized waveforms. Table 2 shows these metrics for both H1A and H1B patients. As seen, the peak-peak amplitude showed the largest error average for both patients with the LATs error being the smallest error average. For the cross-correlation peak and correlation coefficient average error percentages, low correlation coefficients and high cross-correlation peaks can be seen. Both the normalized and original waveforms were used to evaluate amplitude effect as well as general morphology.

**Table 2:** Error and Correlation Values. Error mean and standard deviation values for peak-peak amplitude, deflection count, EGM duration, and LAT across all the signals from both H1A and H1B are shown along with correlation coefficients of electrograms and cross-correlation peak averages for their normalized versions. LATs error were the smallest of all of them meaning that activation of the simulations happened around the same time as the measured EGMs indicated. The largest error was peak-peak amplitude for both patients which probably affected correlation coefficients being low. The average cross-correlation peaks increased substantially after amplitude normalization, highlighting the influence of amplitude on the signal comparison.

	H1A	H1B
Peak-peak Amplitude (% error)	139.76 ± 156.61	192.72 ± 277.28
Deflection Count (% error)	38.49 ± 24.4	42.64 ± 21.7
EGM Duration (% error)	29.17 ± 13.3	25.38 ± 15.95
LATs (% error)	6.93 ± 5.83	13.16 ± 13.8
Correlation coeff. (original)	0.37 ± 0.21	0.3 ± 0.36
Cross-correlation (normalized)	0.85 ± 0.06	0.86 ± 0.14

### 3.2 Principal component analysis on EGMs

**Fig 3:**
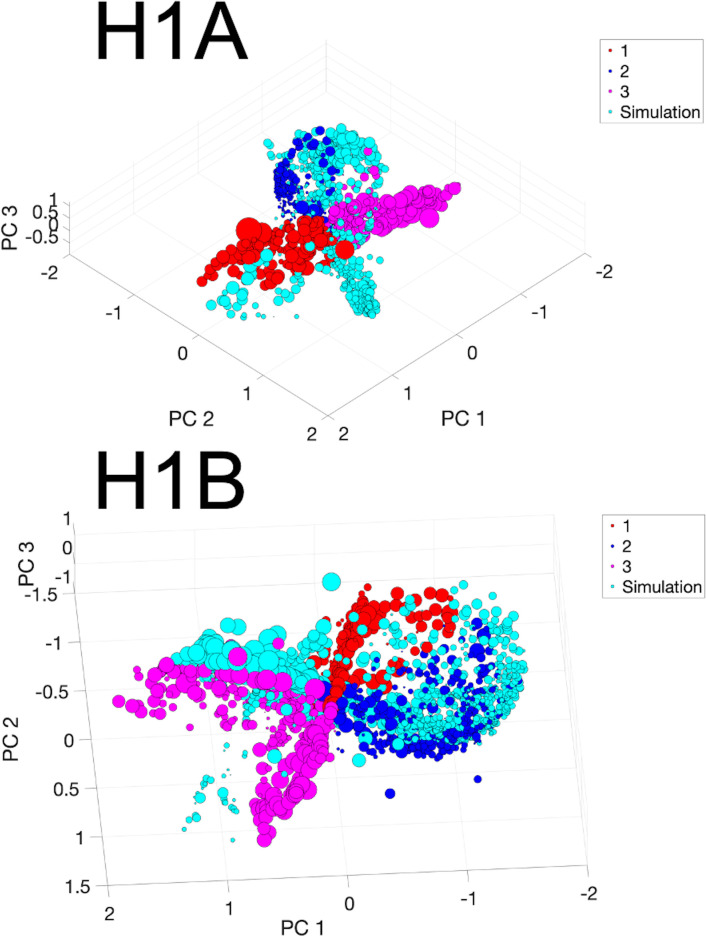
Patients H1A and H1B Principal Component Space. Measured and simulated signals’ PCs coming from patient H1A (left) and H1B (right) are shown using detail coefficients coming from a fourth-order wavelet decomposition on all the measured waveforms of the patients. These PCs coming from measured EGMs were clustered into three different clusters while their eigenvectors were used to map the simulated signals in the same PC space (cyan color). The axes show the first three PCs while the size of each data point corresponds to the fourth PC. Via clustering and visualization, simulated signals fell within the measured signals PCA space. Some of these points lied very closely to other measured signal points, meaning that they simulated some of the signals within the clusters well. Other points lie farther from the clinically measured signals cluster, meaning that those points probably differed more in morphology compared to the measured signals. The models’ performance in simulating EGMs using the detail coefficients metric could be visualized graphically using PCA.

**Fig 4:**
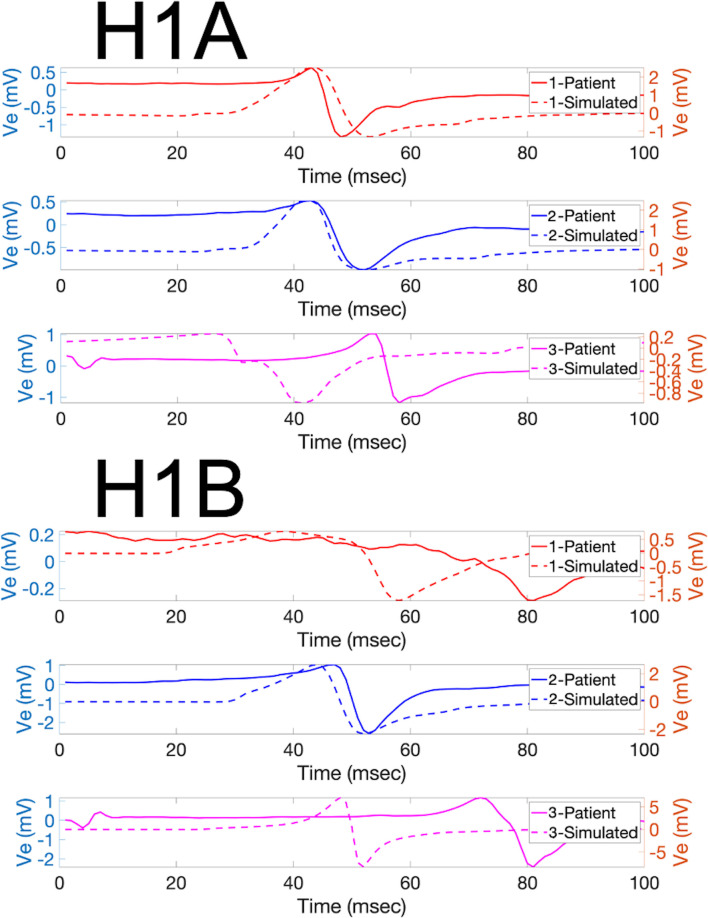
Patients H1A and H1B Sample Clustered Signals. Sample signals (both measured and corresponding simulated) are shown from the different PCA clusters for patients H1A (left) and H1B (right). Even though for PCA generation the normalized signals were used due to the focus on morphology, the signals shown are in mV. The x-axis represents time in msec, and the y-axis represents the extracellular potential in mV, with the left axis corresponding to the measured signal and the right axis to the simulated signal. Two different axes were used to aid in the morphology comparison, since different amplitudes can hinder visual interpretation. The colors correspond to the PCA clusters shown in Fig 3. As observed, some components of the signals were very similar (e.g., the blue signals probably had the same downstroke time). The blue signals even exhibit nicely rounded deflections. Some other components, such as the amplitudes, were not as well captured. The activation times of the pink lines, for instance, differed greatly from one another.

To evaluate the performance of the model from a more graphical perspective, PCA and clustering were used. Using this approach, the fitting of the simulated EGMs in the recorded EGMs PCA space was evaluated. The k-means clustering algorithm utilized three clusters to showcase the groups of signals in those clusters. Fig 3 shows the PC spaces containing the first four PCs for both patients H1A and H1B (x, y, z axes, and size of point). Fig 4 shows randomly selected sample waveforms (both recorded and simulated) from each cluster of the PC spaces seen in Fig 3 which were color-coded according to the cluster color. As seen, the simulated waveforms PCs fit in the PC space of the recorded EGMs mostly well. Interestingly, morphology differences between clusters can be seen in Fig 4. The left axis of the plots in Fig 4 corresponds to the potential (in mV) for the measured waveforms, while the right axis of the plots corresponds to the potential in mV for the simulated waveforms.

Using the PCA spaces seen in Fig 3, differences between the simulated and recorded waveforms’ PC distance-to-centroid. These results can be seen in Table 3. The calculation of the distance-to-centroid showed the spread of the EGMs PCs within each cluster based on the distance values of each signal’s PC point to the centroid of the cluster it “belonged” to for the recorded EGMs. Two different distance values were calculated for the simulated waveforms: the distance from each simulated waveform PCs to its corresponding measured waveform counterpart’s cluster centroid, and the distance value of each waveform PCs to the cluster centroid that was the closest (not necessarily the same as the recorded EGM counterpart). This disparity showed that many times a simulated waveform PCs did not belong to the same cluster that its recorded counterpart waveform belonged to (more than 50% of the waveforms as seen). The distances to the “common” centroids for the simulated signals PCs were larger (between 5 and 9 times larger) compared to the measured signals’ counterparts.

**Table 3:** PCA-based Model Performance. Information about the PCA space distance-to-centroid for the recorded and simulated EGMs PCs is shown. The average PC distance from each recorded EGM PCs to its cluster centroid was calculated, as well as the distance-to-centroid for each simulated EGMs PCs to its corresponding measured waveform counterpart cluster centroid (“common”) and the actual closest cluster centroid of the simulated waveform (“closest”). Because of the disparity between common and closest cluster centroids, the percentage at the end shows the fraction of EGMs whose simulated counterpart belonged to a different cluster; as seen, more than 50% of the simulated EGMs belonged to a different cluster than their recorded counterparts. Simulated EGMs distance-to-centroid values were significantly larger than their measured counterparts even in the closest cluster cases.

	H1A	H1B
Mean Distance to Centroid (Patient)	0.23 ± 0.24	0.25 ± 0.22
Mean Distance to “Common” Centroid (Simulation)	1.17 ± 0.56	1.14 ± 0.55
Mean Distance to “Closest” Centroid (Simulation)	0.79 ± 0.31	0.66 ± 0.32
Different Centroids (%)	60.89	57.66

### 3.3 EGMs isosurfaces

**Fig 5:**
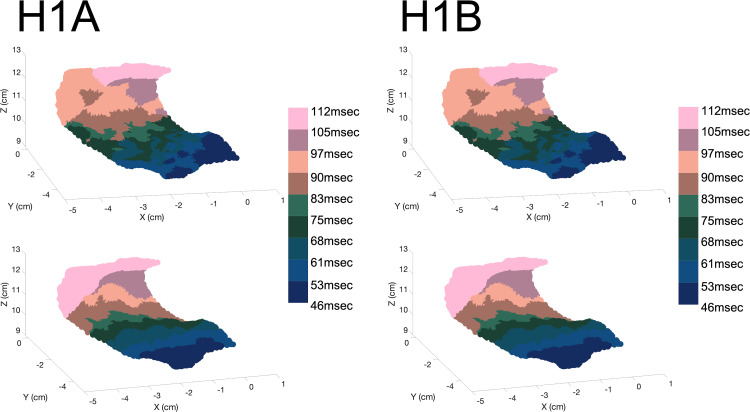
Patients H1A and H1B Isosurfaces. Isosurface maps on all surface points are shown for patients H1A (left) and H1B (right) usin LAT mapping based on electrode proximity for recorded (top) and simulated (bottom) signals. Colorbars show the limits for each surface. The axes are x, y, and z in space. The simulated signal isosurfaces have smoother transitions between LAT ranges compared to the recorded signals. In contrast, the recorded signal isosurfaces had many patches of differing LATs. Even though many points and regions coincided between simulated and recorded data, the recorded EGMs isosurfaces suggest abnormal propagation patterns, as activation occurs in an irregular manner.

**Fig 6:**
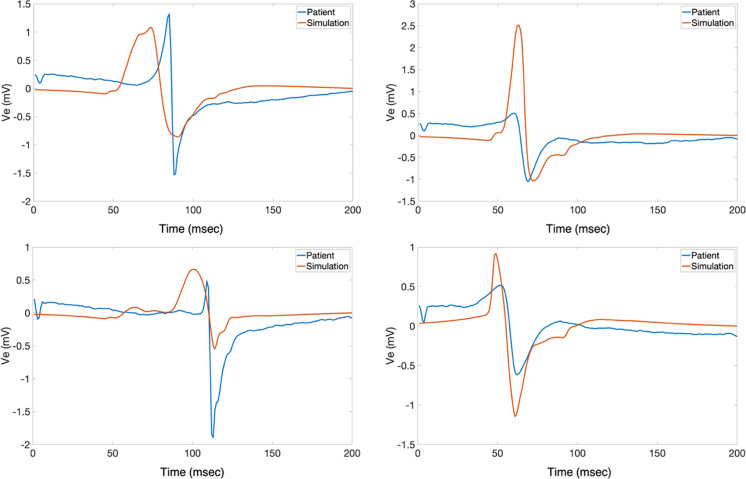
H1A Waveforms. Sample of random signal pairs are shown for points of interest seen in Fig 2 for patient H1A. Blue and orange lines represent recorded and simulated signals, respectively. The x axis represents time in msec, and the y-axis represents electric potential in mV. As seen in the plots, some characteristics of the measured signals were mostly correctly simulated with the models (i.e., timing) while others were not fully captured like amplitudes.

**Fig 7:**
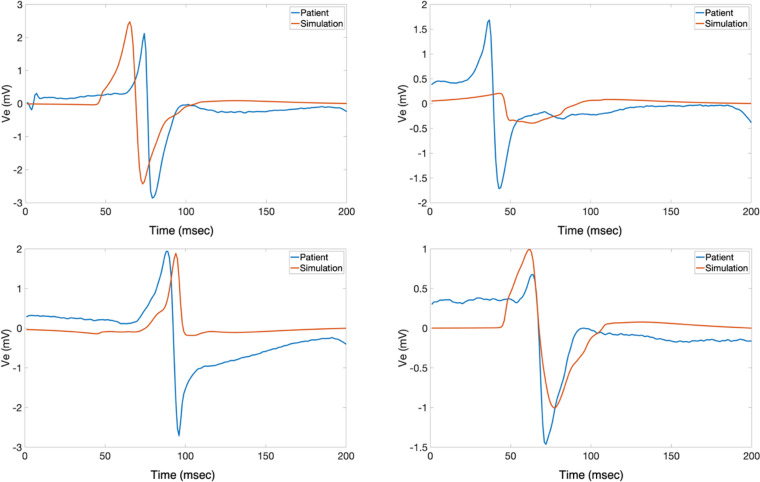
H1B Waveforms. Sample of random signal pairs are shown for points of interest seen in Fig 2 for patient H1B. Blue and orange lines represent recorded and simulated signals, respectively. The x axis represents time in msec, and the y-axis represents electric potential in mV. As seen in the plots, the waveform morphologies in patient H1B seem to be more similar compared to patient H1A. The timings were different, but the characteristics looked better. This comparison can be seen in Tables 1 and 2 as well.

Patients H1A and H1B were used to evaluate the models with healthy tissue (no fibrosis). The LAPW surface points were obtained via fast anatomical mapping while the EGMs and the coordinate points where they were taken were recorded afterwards not via fast anatomical mapping. The results of the mapping made up the PCD LAPW surface points used to create the models, whereas the EGM recordings gave the recorded waveform traces and their locations in space used to validate the models. From the electrodes’ data, the LATs were mapped into each LAPW surface point by obtaining its closest electrode’s LAT. Fig 5 shows the isosurfaces for patients H1A and H1B using the PCD surface points and EGMs LATs. The colorbars show the range limits representing the LATs ranges. Finally, Figs 6 and 7 show samples of randomly selected recorded-simulated waveform pairs from patients H1A and H1B. These EGMs correspond to the blue dots shown in Fig 2 which were randomly selected based on their location in reference to the tissue (i.e., beginning of propagation, end of propagation, middle of the tissue, and close to a region with changing fiber directions). Waveform similarities and differences can be seen when visually inspecting the amplitudes, durations, and general morphologies.

### 3.4 Exploration using fibrotic tissue substrates

**Fig 8:**
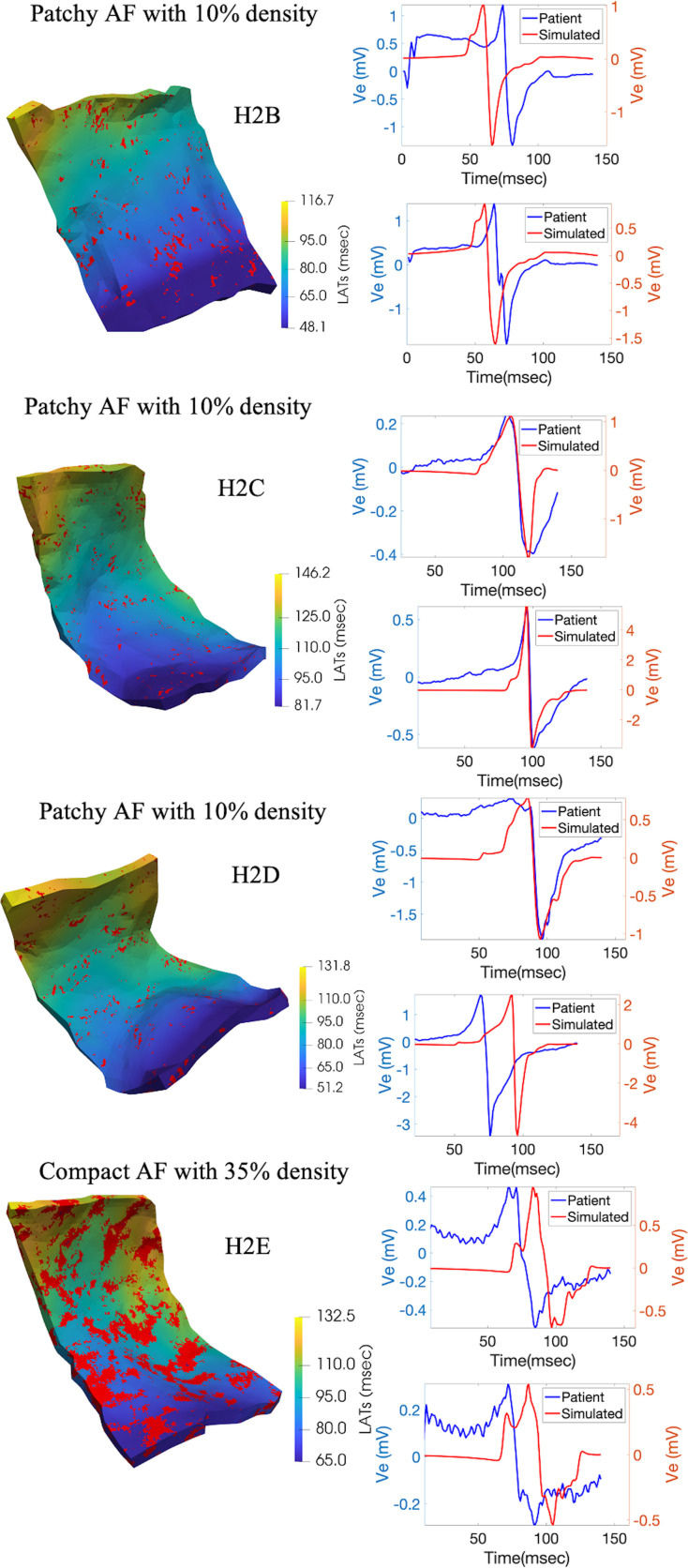
Fibrotic Models Samples with Sample Random Signal Pairs. Fibrotic tissue and recorded-simulated electrogram signals pair for sample fibrotic patterns for the four different model geometries used in fibrotic simulations. Different fibrotic patterns for four different patient-specific models (H2B, H2C, H2D, and H2E) are shown with the resulting local activation times map (color-coded according to colorbar). Sample signal pairs of recorded and simulated electrograms are also shown to display model performance in simulating some of the electrogram waveforms recorded in the clinic. As seen, some components are nicely captured (i.e., general morphology, some activation times) while other components were not fully obtained (i.e., amplitude). These waveforms were characterized for all simulations to obtain Table 4.

To explore the effect of atrial fibrosis on EGM morphology and characteristics, PCD models of four different patient geometries were used with different fibrosis configurations. These models allow us to evaluate characteristics and differences in EGMs with the different configurations prescribed (combinations of different atrial fibrosis types and degrees) using the models generated for each patient. Fig 8 contains a sample pattern for the four patient-specific models used mapped on top of the LATs map in the tissue subdomain. Sample random EGMs for each of those patient model simulations are shown as well; these EGMs along with the other 476 patterns simulations were characterized to compare EGMs with different atrial fibrosis types and degrees. To evaluate this parametric variability, Table 4 shows the average and standard deviation of some of the characteristic parameters across all patient models and random patterns divided by fibrosis type and degree. The values shown are not just average values for all the EGMs in a single given configuration simulation but also across the ten different simulations of all combinations of different types and degrees of atrial fibrosis for each patient model (all 480 simulations). In this way, the overall variability of the characteristics coming from simulated EGMs across patients, fibrosis type, and fibrosis degree could be assessed. These results suggest that the major determinant for how the parameters varied across categories was the degree of atrial fibrosis and not its type. To confirm the statistical significance of these differences observed, one-way ANOVA tests were performed to evaluate the differences in the mean values for each of the 12 combinations of fibrosis type and degree for all patient models and patterns [73]. Table 5 shows that fibrosis percentage (10%, 35%, 60%) had a strong and consistent effect on electrogram features across all morphologies (F = 5,562–68,087, p < 0.0001), with Tukey’s post-hoc confirming significant differences between all percentage pairs [74]. Fibrosis type (diffuse, compact, patchy, interstitial) also produced significant differences at fixed percentages (F = 16.7–3,064, p < 0.0001), though some comparisons (e.g., diffuse vs interstitial at 10% and 35%) were not significant. The much larger F values for fibrosis percentage indicate it is the dominant factor affecting electrogram changes, with morphology having a secondary, less consistent effect.

**Table 4:** Simulated EGMs Characteristics Variability (Atrial Fibrosis Degree and Type). The table shows the characteristic parameters variability across the combinations of different types and degrees of fibrosis. For each combination, the parameters are given in average and standard deviation values. From the table, it can be seen that the differences between the characteristics are more sensitive to fibrosis degree compared to fibrosis density. For instance, the mean and standard deviations of EGM durations and deflection counts seem to have increased as the fibrosis density increased, but the mean and standard deviation of the peak-peak amplitude values seem to decrease with increasing fibrosis density. On the other hand, there is no significant distinction in characteristic parameters values between types of fibrosis for all the fibrosis densities. These observations were confirmed with the statistical test done and shown in Table 5.

		Compact atrial fibrosis	Diffuse atrial fibrosis	Interstitial atrial fibrosis	Patchy atrial fibrosis
10%	EGM Duration (msec)	73.77 ± 15.54	73.45 ± 15.12	73.34 ± 14.97	73.68 ± 15.27
	Peak-peak A. (mV)	2.8 ± 3.01	2.99 ± 3.16	3 ± 3.18	2.9 ± 3.11
	Deflection Count	4.07 ± 1.61	3.75 ± 1.53	3.69 ± 1.54	3.84 ± 1.55
35%	EGM Duration (msec)	77.17 ± 18.2	76.39 ± 18.7	76.53 ± 17.54	76.68 ± 17.73
	Peak-peak A. (mV)	1.59 ± 1.77	1.98 ± 2.21	1.86 ± 2.09	1.73 ± 1.89
	Deflection Count	5.52 ± 2.26	4.49 ± 2	4.22 ± 1.84	4.82 ± 2.04
60%	EGM Duration (msec)	80.59 ± 20.47	84.89 ± 21.66	86.35 ± 20.04	83.89 ± 19.5
	Peak-peak A. (mV)	0.55 ± 0.81	0.56 ± 0.81	0.49 ± 0.65	0.52 ± 0.67
	Deflection Count	7.49 ± 2.95	7.22 ± 3.19	6.99 ± 2.99	7.46 ± 3.03

**Table 5:** One-way ANOVA and Tukey’s Significant Differences. The table shows the results of the statistical test performed on the differences between EGM duration, Peak-to-peak amplitude, and deflection count metrics across the different fibrosis simulation groups. The results demonstrated that fibrosis percentage (10%, 35%, 60%) had a consistently strong effect on electrogram features across all fibrosis morphologies (F = 5,562–68,087, p < 0.0001), with Tukey’s post-hoc confirming that every pair of percentages was significantly different. In contrast, while fibrosis type (diffuse, compact, patchy, interstitial) also produced significant differences at fixed percentages (F = 16.7–3,064, p < 0.0001), certain morphologies (e.g., diffuse vs interstitial at 10% and 35%) did not differ significantly. Even though the different fibrosis types produced significant differences, the much higher F-statistic values for differences in fibrosis degree compared to fibrosis type indicate that fibrosis percentage is the dominant determinant of electrogram alterations, with morphology exerting secondary, less consistent effects.

Comparison type	Metric	F-statistic	p-value	Tukey post-hoc significant differences (α = 0.05)
Within Diffuse (10,35,60%)	EGM Duration	12636.8	<0.0001	All pairs
	Deflection Count	42752.5	<0.0001	All pairs
	Peak-peak Amplitude	5562.4	<0.0001	All pairs
Within Compact (10,35,60%)	EGM Duration	11540.9	<0.0001	All pairs
	Deflection Count	32166.8	<0.0001	All pairs
	Peak-peak Amplitude	6354.9	<0.0001	All pairs
Within Patchy (10,35,60%)	EGM Duration	16891.6	<0.0001	All pairs
	Deflection Count	49762.7	<0.0001	All pairs
	Peak-peak Amplitude	6552	<0.0001	All pairs
Within Interstitial (10,35,60%)	EGM Duration	19930.9	<0.0001	All pairs
	Deflection Count	68086.7	<0.0001	All pairs
	Peak-peak Amplitude	6715	<0.0001	All pairs
Across fibrosis types (10%)	EGM Duration	37.6	<0.0001	All pairs except diffuse vs interstitial
	Deflection Count	3064	<0.0001	All pairs
	Peak-peak Amplitude	16.7	<0.0001	All pairs except diffuse vs interstitial
Across fibrosis types (35%)	EGM Duration	129.4	<0.0001	All pairs except diffuse vs interstitial
	Deflection Count	1399.4	<0.0001	All pairs except diffuse vs interstitial
	Peak-peak Amplitude	138.9	<0.0001	All pairs
Across fibrosis types (60%)	EGM Duration	130.3	<0.0001	All pairs except compact vs patchy
	Deflection Count	498.1	<0.0001	All pairs except compact vs diffuse
	Peak-peak Amplitude	97.1	<0.0001	All pairs

The table shows the results of the statistical test performed on the differences between EGM duration, Peak-to-peak amplitude, and deflection count metrics across the different fibrosis simulation groups. The results demonstrated that fibrosis percentage (10%, 35%, 60%) had a consistently strong effect on electrogram features across all fibrosis morphologies (F = 5,562–68,087, p < 0.0001), with Tukey’s post-hoc confirming that every pair of percentages was significantly different. In contrast, while fibrosis type (diffuse, compact, patchy, interstitial) also produced significant differences at fixed percentages (F = 16.7–3,064, p < 0.0001), certain morphologies (e.g., diffuse vs interstitial at 10% and 35%) did not differ significantly. Even though the different fibrosis types produced significant differences, the much higher F-statistic values for differences in fibrosis degree compared to fibrosis type indicate that fibrosis percentage is the dominant determinant of electrogram alterations, with morphology exerting secondary, less consistent effects.

## 4 Discussion

A total of six PCD models were generated accounting for six different patients. Simulated EGMs were obtained via the forward calculation utilizing transmembrane potentials modeled using the monodomain equations and the Courtemanche model. A key step in model construction was the initial downsampling of the original PCD containing the atrial surface points. Downsampling led to smoother surfaces that were more easily extruded and eventually to models that generated simulated EGMs comparable to recorded waveforms. Downsampling the PCD for the surface points (not the electrode points) allowed the correct surface generation, which would have been more difficult without downsampling because extrusion would result in overlapping surfaces. With this simple but robust pipeline, creating PCD models took less than thirty minutes in the author’s experience, which has the potential for other applications requiring fast model generation methodologies. This methodology of model has the potential to solve the propagation problem, with most of these simulations taking between one and two and a half hours to finish using at least 220 cores. The majority of this time was spent in assembling matrices, setting fiber orientations, and setting fibrosis domains, because these steps were not parallelized, unlike the solution of the system itself.

As shown in the top right panel in Fig 7, considerable waveform morphology differences were found in electrodes close to drastically changing fiber orientation fields. These differences could be caused by the fiber orientation mapping used. In fact, the template mesh contains the general directions of the muscle fibers, but it cannot account for peculiarities that patients might have in their tissue substrates. More studies would need to be done with different fiber fields to evaluate the effect of them on the resulting morphologies. Differences between simulated and recorded isosurfaces created from the LATs were likely due to the unequal spatial distribution of electrode points above the surface points. As shown in Table 2, the LATs average error for patient H1A was 6.93% and the error for patient H1B was 13.16%. To more accurately estimate LATs in the tissue, which generate isosurfaces, enough electrograms need to be recorded. Recording enough electrograms to cover the whole range of the tissue region being studied is key to understanding fully what is happening in that region, which is typically not needed for clinical procedures due to minimization of invasiveness to the patient. The fact that the LATs surface map does not contain accurate times could have arisen due to the spatial differences in electrode recordings for both patients (i.e., waveform recordings done with different proximities to the tissue in the tissue normal direction as well as along the fibers).

**Fig 9:**
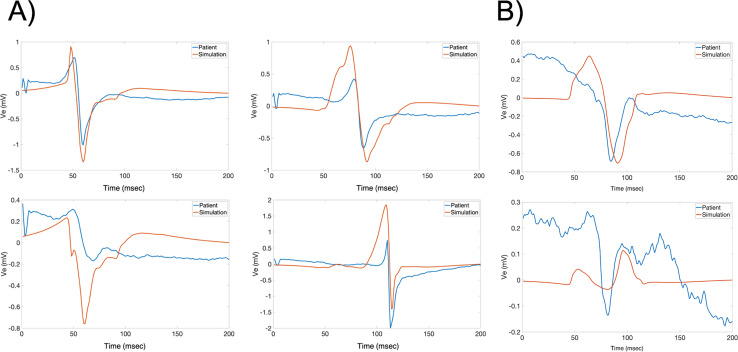
Additional Sample Waveforms. A) Additional randomly selected electrode points based on locations are shown: towards the beginning of propagation (top left), towards the middle of the tissue domain (top right), close to tissue curvature (bottom left), and towards the end of propagation (bottom right). Blue and orange lines represent the recorded and simulated traces, respectively. The x and y axes are time (msec) and amplitude (mV), respectively. Amplitude and morphology similarities are shown, while also the presence of more deflections in healthy tissue configuration than a biphasic signal is shown. B) Sample signals for patient H1B are shown selected based on location: from an electrode point right before the side edge of the geometry (top) and an electrode point past the edge of the geometry (bottom). Blue and orange lines represent the recorded and simulated traces, respectively; the x and y axes are time (msec) and amplitude (mV), respectively. Far-field activity happening in the recorded signals was not seen in the simulated signals as shown.

Besides morphology and time, the general pattern of activation is worth analyzing in both recorded and simulated EGMs. Localized LATs “patches” containing points of differing LATs compared to the surroundings arise only in the recorded EGM surfaces and not in the simulations. In the simulated EGM isosurfaces, the pattern of activation is more sequential with smoother LATs ranges and edges. This phenomenon could be explained by paroxysmal AF patients having some degree of fibrosis causing abnormal pathways and therefore localized delays that could lead to different LATs within a region. It is generally accepted that persistent AF patients experience more remodeling or fibrogenesis than paroxysmal AF patients [75,76], and the abnormal LATs patches in the recorded EGM isosurfaces could indicate that there might be sufficient remodeling to create these abnormal pathways. The morphologies of waveforms in Figs 6, 7, and 9 also hint to these potential differences arising due to fibrosis since fractionation bumps are apparent in parts of the traces.

Other possible reasons for the differences could be attributed to modeling artifacts. These artifacts rising from modeling methodologies could be tissue and blood bath properties, but it also includes limitations of modeling. Fig 9A shows patient H1A randomly selected waveform pairs located at: the beginning of propagation (top left), towards the middle of the tissue (top right), near tissue curvature (bottom left), and towards the end propagation (bottom right). There are comparable characteristics, but it is interesting to see deflections arise near curved regions since the tissue substrate is non-fibrotic. The simulated EGM shows an extra deflection around the time of activation (during the downstroke) that is absent in the recorded EGM. At the same time, Fig 9B shows EGMs coming from electrode points close to the edges of the domain. In the clinical recordings, there could have been far-field activity that affected the recorded EGMs morphologies that was not accounted for in the local model.

As seen in Figs 3 and 4, the simulated EGMs fall within the PCA space of the recorded EGMs, but there are some important differences. As Table 3 shows, the simulated EGM PCs lied further out from the cluster centroids compared to their recorded EGM PCs counterparts. The distance was even greater when calculating the distance of the simulated EGM PCs to the cluster of its recorded EGM PCs counterparts. With more than 50% of simulated-recorded EGM pairs not belonging to the same cluster, the PC analysis indicated that the simulated EGMs had some significant differences.

The comparison between measured and simulated electrograms reveals important insights into the model’s accuracy and its ability to replicate patient-specific electrophysiological characteristics. While most parameters fell within the expected numerical range as seen in Table 1, some discrepancies remained, particularly in the signal width for patient H1A. These differences suggest that certain aspects of the simulation may not fully capture patient-specific variations, potentially due to differences in tissue properties and atrial substrate or unaccounted physiological factors. By assessing the effect size using Cohen’s d values, we can better understand the practical significance of these discrepancies rather than just their statistical presence. Interestingly, patient H1B model exhibited smaller effect sizes in most characteristic parameters, suggesting a better agreement between simulated and measured signals compared to patient H1A. However, despite H1A showing larger effect sizes in some parameters, it still outperformed H1B in LATs and amplitude errors, raising the question of whether certain model assumptions favor specific features over others. These findings highlight the need for further refinements in model calibration and parameter tuning to improve agreement across all key characteristics while highlighting the importance of understanding the atrial substrate state beyond just the correct geometry. The latter is a challenging thing to achieve without more invasive procedures like histological sampling or high resolution imaging. Additionally, the observed discrepancies may provide insight into underlying electrophysiological differences between the patients, which could be further explored in future studies.

In H1 patients, simulated EGMs showed high peak-to-peak amplitude errors and notable PCA cluster mismatches. The amplitude discrepancies likely stem from the choice of bath conductivity parameter ($\sigma_{b}$) in the extracellular potential calculation, which acts as a scaling factor when assuming blood acts as a homogeneous isotropic volume conductor as seen in Equation 5 [47]. It is well-known that the tissue and extracellular conductivities giving rise to the functional monodomain conductivity tensor governs action potential (AP) propagation, influencing CV and the spatial-gradients of $\begin{array}{c} V_{\text{m}} \end{array}$ which make EGMs sensitive to the local activation times and shifting signals in time [77,78]. This effect means that choosing higher or lower values for these conductivities would create higher or lower CV values, respectively, affecting timing of EGMs. On the other hand, blood bath conductivity in monodomain simulations does not influence AP propagation but only the retrieval of extracellular potentials, making the EGM amplitude inversely sensitive to its magnitude. It has been shown that bath does influence AP propagation in the form of increasing wavefront curvature [47], but in pure monodomain models this effect is not taken into account. Due to this higher effect in amplitude by the bath conductivity, the EGM amplitude discrepancies were mostly sensitive to blood conductivity than the functional monodomain conductivity; choosing a higher or lower value would decrease or increase the amplitude, respectively. Addressing this discrepancy for better fit would require parameter estimation approaches bounded by reported ranges [79] or more detailed models (e.g., pseudo-bidomain or bidomain) that were beyond this study’s scope. PCA mismatches, despite normalization, reflect differences in morphology-related features such as LATs, APD, and deflection count. These limitations suggest that while the current approach is not optimal for applications requiring exact morphology reproduction, it remains suitable for investigating macro-scale conduction patterns and electrophysiological behavior as well as comparable amplitude values if extra tuning is performed.

As for the exploratory study on usability of these models with fibrotic substrates, three of the characteristic parameters of all the substrate configurations grouped by type and degree can be seen in Table 4. Clear differences between peak-peak amplitude and deflection count across the different degrees of fibrosis could be appreciated, but there was no significant differentiation between different types of fibrosis at any fibrosis degree. This significance of the differences between the groups was confirmed through our analysis of variance test and Tukey’s Significant Differences test as it can be seen in Table 5. Because of the large number of measurements obtained through the simulations, the F-statistic values were very large for differences between fibrosis degrees. For the differences between fibrosis types, the F-statistic values were also large but not as large as the fibrosis degree differences, indicating a higher effect of fibrosis degree compared to fibrosis type. Interestingly, at higher densities, such as in the 60% case, the differences between atrial fibrosis types are less obvious. The EGM duration changed more as atrial fibrosis degree increased, but there was not a significant difference in the average value between 10% and 35% atrial fibrosis density for any type of fibrosis. The parameters EGM duration and deflection count seemed to increase, but peak-peak amplitude went down with increasing atrial fibrosis density. This seen behavior is in line with previous work that shows that for the particular fibrosis modeling methodology that was used smaller peak to peak amplitudes and higher fractionation is seen in simulated EGMs [80]. With models behaving as expected, the usage of such PCD-generated models can therefore be used for more complex studies. The trends observed suggest the higher relevance of atrial fibrosis density compared to its type. Understanding the relationship between atrial fibrosis type and density to EGM morphology and characteristics can improve interventions in the clinic based on the important biomarkers while discarding the less important ones. Nonetheless, one of the limitations of this study was the sample size of persistent AF patients that had usable PCD geometries, therefore a more in-depth exploration on fibrosis degree vs pattern could be made with a larger sample size. Our study quantifies the general trends of the differences given that we had 480 different combinations of patterns, but having more than four patient specific geometries could help explore other geometries that could provide more insight on these trends.

**Fig 10:**
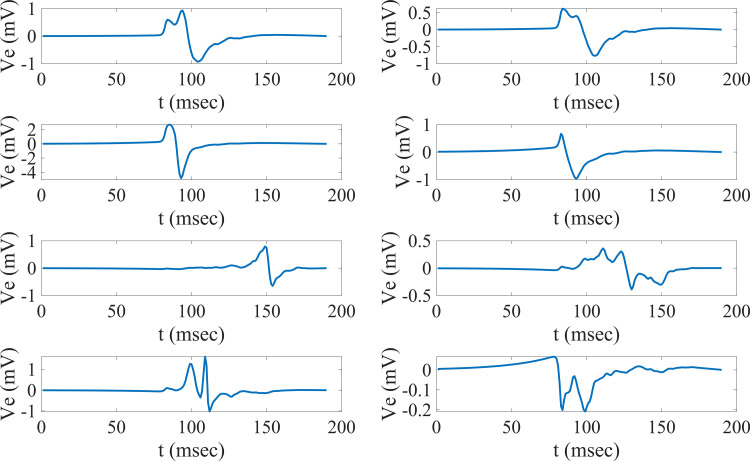
Sample Waveforms from Fibrosis Simulations. Selected sample waveforms with morphologies comparable to the computational study done by Campos et al. Campos and colleagues simulated patterns with uncoupled tissue with orientations parallel to muscle fibers (similar to interstitial or patchy fibrosis) and orientations with multiple random directions (similar to diffuse fibrosis). Since in their work they had these densities as mid-level and higher-level fibrosis densities, sample waveforms are shown for fibrosis simulations with set-ups more closely similar to the ones from Campos’ work. Electrograms with similar morphologies are shown for these two types of tissues; these electrograms come from one of the simulation configurations with 35% interstitial fibrosis and another simulation configuration with a 60% diffuse fibrosis. As seen, morphological characteristics like deflection patterns and amplitudes are comparable to the results from Campos et al., speaking to the ability of PCD models to generate waveforms comparable to published studies.

Even though the focus of the study was a simulation-clinical recordings comparison, the EGMs coming from PCD models were also compared to waveforms obtained from state of the field models. In this way, PCD models could not only be validated using the recorded waveforms but also validated by comparison to published data from other models. A relevant study that took into account fibrosis and waveform morphology was the one done by Campos et al. [81]. This study focused on the influence of the angle at which the wavefront propagation approaches an electrode tip close to fibrotic tissue. Even though the specific patterns used in this study were not used in our study, comparable waveforms can be seen from the models with interstitial and diffuse fibrosis. Fig 10 shows waveforms coming from a simulation with interstitial fibrosis with 35% density (top four EGMs) and waveforms coming from a simulation with diffuse fibrosis with 60% density (bottom four EGMs). These waveforms observed from the simulations have comparable morphologies and characteristics to the ones shown by Campos and colleagues in the groups trying to replicate interstitial or patchy fibrosis and diffuse fibrosis. Deflection locations were comparable (i.e., two positive deflections before a large negative deflection or a spike in the middle of the downstroke) as well as similarities in amplitudes (i.e., amplitudes between 1 and 2 mV for the majority of the simulations shown). The goal was not to match patterns or waveforms but to evaluate how the morphologies and characteristics compared to the state-of-the-art models and EGMs; interestingly, even without achieving a perfect fibrosis pattern match, results are comparable, stating more strongly the possibility of PCD models to be more widely used to generate waveforms that compare to other models’ waveforms and recorded waveforms. These comparable results speak to the potential of using the presented model construction pipeline for electrophysiology studies focusing on EGMs.

Besides comparing the simulated EGMs to the recorded counterparts as well as what the state of the field is, it is worth comparing the model generation methodology itself with other model generation workflows. Recently, two automated pipelines have been developed to streamline the creation of patient-specific atrial models from clinical data. For example, the AugmentA pipeline, introduced by Azzolin et al., generates ready-to-use computational models from atrial surfaces derived from either MRI/CT imaging or electroanatomical maps [82]. Similarly, the atrial modelling toolkit (atrialmtk) by Bevis et al. uses segmentation masks from CT or MRI images as input to create simulation-ready meshes [83]. These frameworks are designed to produce comprehensive, anatomically detailed models, often incorporating features like fiber orientation, anatomical region annotation, and transmural variations across the entire atrium. AugmentA, for instance, uses a statistical shape model (SSM) to regularize anatomy and augment missing structures, such as the right atrium or appendages, and validates its models by comparing simulated and clinical local activation time (LAT) maps. The atrialmtk pipeline also offers multiple workflows to handle various input data types and can map atrial structures and fiber distributions from atlases onto the patient’s anatomy.

A significant strength of our methodology is the computational efficiency in storage when compared to image-based approaches. For instance, images coming from MRI data used form segmentation can require multiple GB’s for a single case [84], while a .MAT file with PCD for the whole atria (not even the trimmed points for the LAPW only) along with the waveform time traces for a single case was less than 5MBs. This memory efficiency not only facilitates the handling of multiple PCD data files but also the computational power required to process a case in software. When doing image segmentation, powerful machines can be needed to process MRI data successfully [85], but when handling PCD since the data is so compact the computational resources needed are much less. The PCD mesh generation for the models in this work were done on a MacBook Pro Laptop with a 2.4 GHz Quad-Core Intel Core i5 and 8 GB of RAM. Besides the computational efficiency component, image segmentation typically requires an expert doctor to spend between 15–20 minutes on specialized software cleaning and creating the image segmentation that is then used as the input to the rest of the mesh generation pipeline. This time and complexity is due to image resolution limitations (affecting the detection of muscle wall sometimes) as well as the manual clean up of the segmentation and manual threshold corrections due to the atrial blood pool [43,86–88]. With the pipeline discussed in this work, the finalized patient-specific mesh without the fiber architecture would take at most 30 minutes with the more complex cases and around 10 minutes with the less complex LAPW cases. The steps that were used in the pipeline can be summarized in the following: LAPW point coordinates extraction (around 1 minute), downsampling and surface mesh creation using the LAPW PCD (around 1 minute), surface vertices clean up and extrusion using Blender (between 5 and 20 minutes since it was mostly a manual process and was affected by overlapping surfaces), and volumetric mesh creation using Coreform Cubit (around 2 minutes). Unlike comprehensive pipelines such as AugmentA and atrialmtk, which often require multiple dependencies like openCARP and interactive landmark selection in separate software, our approach creates geometrically simpler models that strictly adhere to the patient’s recorded point cloud data. Furthermore, we introduce a different validation endpoint: whereas Azzolin et al. focused on replicating LAT maps, our validation included LAT error calculation as well morphological characterization comparison of simulated EGMs to clinical recordings. The pipeline used in this work had the limitation of working on surface such as the LAPW, which is a very important part of the atrial arrhythmogenesis, but it does not represent the whole atrium. When specific localized studies focusing on specific walls and components of the cardiac architecture are the goal, using PCD models can be very beneficial and electrophysiology studies.

The performance of the models was evaluated using different numerical and graphical approaches. The LATs, which is a parameter of high importance for clinicians when evaluating the state of a patient, had small errors when comparing simulated vs recorded EGMs [89]. On the other hand, characteristics that are relevant for clinicians like the amplitude [90,91] had errors that were very high. Generated waveforms seem to compare to other simulated results published in the literature, and the model generation methodology used seems to provide benefits in comparison to other approaches. Even though the study was limited to LAPW geometries, this methodology proves to be useful in studying EGMs coming from patient-specific simulations from PCD models. More challenging processing of PCD’s would need to occur to create PCD models capable of handling more complex geometries; nonetheless, simulations with LAPW PCD models can provide valuable insights when studies focused on region-specific dynamics. For instance, *in silico* studies on AP propagation dynamics influenced by changes at the cellular and ion channels level performed typically using tissue slabs could benefit from evaluating their metrics using patient-specific models instead [6,92,93]. Extending tissue slab studies of cellular-to-macro level dynamics into using realistic three-dimensional models of the LAPW where many reentrant activities start especially in persistent AF patients [75,76] can provide new insights on drug and therapy studies. Overall, PCD models of the LAPW showed to be useful in studying arrhythmogenic behavior and patient-specific conditions.

## 5 Conclusions

This work aimed to utilize patient data obtained from EAMS to create patient-specific models from PCD that could generate simulated waveforms and characteristics comparable to recorded ones and to use these models to explore atrial fibrosis effect in EGM morphology. A total of six PCD models were generated and successfully reached convergence in the solution of electrophysiology equations. The waveforms and their characteristic parameters coming from these simulations were compared to clinical patient data. Some waveform characteristics could be replicated for two paroxysmal AF patient EGMs using healthy tissue configurations like LATs, but some other parameters like amplitudes were not replicated correctly, as seen from the different metrics utilized. Isosurfaces showed unexpected propagation patterns for both patients H1A and H1B with the embedded LATs “patches” seen in the maps; these patches could be related to the possible presence of fibrotic obstacles in these areas. In the exploratory study modeling atrial fibrosis, it was seen that characteristic parameters are more sensible to atrial fibrosis density changes than to atrial fibrosis type changes, suggesting its relevance in arrhythmogenesis and EGM fragmentation. The dataset consisting of geometries, signals analyzed, functions, and models generated for this study can be found in a public repository for ease of access and reproducibility (https://doi.org/10.5061/dryad.c866t1gn7).

It is important to acknowledge the limitations of this study related to sample size. The validation of EGM morphology was conducted on only two patient geometries (H1A and H1B). While these cases provide a valuable proof-of-concept, this small cohort restricts the ability to draw generalizable conclusions about the model’s accuracy across a wider patient population. Furthermore, the fibrosis exploration was performed using four geometries (H2B, H2C, H2D, H2E). The statistical power of this sample is insufficient to make definitive claims about the relative impact of fibrosis type versus density. Therefore, while our results are promising, they should be interpreted as preliminary. Future work should aim to validate this methodology on a larger and more diverse patient cohort to substantiate these findings.

Despite improvements in AI-based methods for medical image analysis, model generation using medical image segmentation remains a complex, specialized, and time-consuming job to do without the right training, experience, and knowledge of the heart and its anatomy. Creating patient-specific models from PCD without image segmentation is a step towards generating compact but powerful and memory efficient models that can be used for diagnosis, therapies, and pathophysiology studies. The benefit of using PCD models is that EGMs are obtained during the same anatomical recording procedures, and these EGMs can be used easily for model validation and model performance evaluation. Adaptability and rapid processing capabilities are some of the attractive features of this model generation pipeline. This proof-of-concept methodology for creating useful mathematical meshes from PCD originating from patient data and its explicit step-by-step pipeline is one of the main contributions of this study. Another relevant component of this work is the use of multiple tools to evaluate model performance (i.e., isosurfaces, PCA, etc.) and to compare recorded-simulated EGMs which could be standardized to create platforms that generate and evaluate PCD models based on the data that EAMS already record. PCD models proved to be enough to create LAPW patient-specific models due to the LAPW’s simpler geometry. This sufficiency makes PCD models promising tools in patient-specific modeling.
